# Comparing three approaches for involving patients in research prioritization: a qualitative study of participant experiences

**DOI:** 10.1186/s40900-020-00196-4

**Published:** 2020-05-01

**Authors:** Danielle C. Lavallee, Sarah O. Lawrence, Andrew L. Avins, David R. Nerenz, Todd C. Edwards, Donald L. Patrick, Zoya Bauer, Anjali R. Truitt, Sarah E. Monsell, Mary R. Scott, Jeffrey G. Jarvik

**Affiliations:** 1 Dept. of Surgery, University of Washington, Surgical Outcomes Research Center, 1107 NE 45th St., Suite 502, Seattle, WA 98105 USA; 2 Dept. of Health Services, University of Washington, 1959 NE Pacific St., Seattle, WA 98195 USA; 3 Division of Research, Kaiser Permanente Northern California, 2000 Broadway, Oakland, CA 94612 USA; 4 Henry Ford Health System, Suite 3A, One Ford Place, Detroit, MI 48202 USA; 5 Division of Medical Oncology, University of Washington, 825 Eastlake Ave. E, Seattle, WA 98109 USA; 6 Dept. of Radiology, Comparative Effectiveness, Cost and Outcomes Research Center, University of Washington, 4333 Brooklyn Ave. NE, Seattle, WA 98105 USA; 7 HealthPartners Institute, 3311 E. Old Shakopee Road, Bloomington, MN 55425 USA; 8 Dept. of Biostatistics, University of Washington, 1705 NE Pacific St, Seattle, WA 98195 USA; 9 Dept. Neurological Surgery, University of Washington, 1959 NE Pacific St, Seattle, WA 98195 USA

**Keywords:** Patient involvement, Patient engagement, Patient-centered outcomes research, Qualitative research, Research priorities, Crowd-voting, Focus groups, Delphi survey, Nominal group technique

## Abstract

**Background:**

By participating in priority-setting activities in research, patients and members of the public help ensure that important questions are incorporated into future research agendas. Surveys, focus groups, and online crowdsourcing are increasingly used to obtain input, yet little is known about how they compare for prioritizing research topics. To address this gap, the Study of Methods for Assessing Research Topic Elicitation and pRioritization (SMARTER) evaluated participant satisfaction with the engagement experience across three prioritization activities.

**Methods:**

Respondents from Back pain Outcomes using Longitudinal Data (BOLD), a registry of patients 65 years and older with low back pain (LBP), were randomly assigned to one of three interactive prioritization activities: online crowd-voting, in-person focus groups using nominal group technique, and two rounds of a mailed survey (Delphi). To assess quality of experience, participants completed a brief survey; a subset were subsequently interviewed. We used descriptive statistics to characterize participants, and we analyzed responses to the evaluation using a mixed-methods approach, tabulating responses to Likert-scale questions and using thematic analysis of interviews to explore participant understanding of the activity and perceptions of experience.

**Results:**

The crowd-voting activity had 38 participants, focus groups 39, and the Delphi survey 74. Women outnumbered men in the focus groups and Delphi survey; otherwise, demographics among groups were similar, with participants being predominantly white, non-Hispanic, and college educated. Activities generated similar lists of research priorities, including causes of LBP, improving physician-patient communication, and self-care strategies. The evaluation survey was completed by 123 participants. Of these, 31 across all activities were interviewed about motivations to participate, understanding of activity goals, logistics, clarity of instructions, and the role of patients in research. Focus group participants rated their experience highest, in both the evaluation and interviews.

**Conclusion:**

Common methods for research prioritization yielded similar priorities but differing perceptions of experience. Such comparative studies are rare but important in understanding methods to involve patients and the public in research. Preferences for different methods may vary across stakeholder groups; this warrants future study.

**Trial registration:**

NICHSR, HSRP20152274. Registered 19 February 2015.

## Plain english summary

Asking patients and the public to help design and conduct research is an important next step beyond simply participating in a study. Patient and public involvement in research decisions brings fresh views about using scarce resources to address their specific needs. Too often, only researchers and funding agencies have a say. Yet, the best ways to involve patients in shaping research remain unclear. In this project, patients drawn from a registry of those 65 years or older with low back pain were asked to participate in three possible activities to prioritize research: 1) crowd-voting, where users can connect online but not always in real time; 2) focus groups, where a moderator leads in-person discussions; and 3) a paper-based survey, where interactions are written. Participants evaluated their experiences through a survey and interviews. As predicted, participants preferred the in-person focus group activity best. However, the activity’s purpose was not always clear. Focus groups were thought to be about understanding low back pain, when the goal was to prioritize research topics. Participants in the other two activities (crowd-voting and the survey) were less positive about their experience but understood the purpose better. These and other results show one approach will not fit all. Patients differ in many ways, including age, type of illness, access to health care, and comfort with technology. Understanding how methods compare from the participant’s perspective informs decisions when designing activities to prioritize research with the goal to expand patient and public involvement.

## Background

Involving patients and the public, especially when research topics are formulated, is a cornerstone of patient-centered outcomes research. Firsthand knowledge of a health condition and its impact on day-to-day life often yields research priorities not recognized or reflected by researchers and policymakers alone [1, 2]. For example, in 2000, Tallon and colleagues demonstrated a mismatch in areas of research and stakeholder needs. An evaluation of osteoarthritis studies found the bulk of research focused on drugs and surgical interventions. In contrast, focus group discussions with patients identified a need for evidence across treatment options (e.g., physical therapy, injections, complementary therapy, etc.) and effective education strategies [1]. This discrepancy highlights the need for contributions from patients and the public as research priorities are determined.

Funding agencies, governments, and advocacy organizations seek to better align research agendas with the needs of stakeholders by employing diverse methods to involve them in priority-setting activities [3–6]. Methods include surveys, focus groups, consensus-building activities, and, more recently, crowdsourcing [7]. Such efforts provide opportunities for understanding the interests, needs, and perspectives of stakeholders in order to improve the value and quality of evidence generated [8]. Little is known, though, about how different methods compare for priorities generated and participant experience. Evidence that directly compares different patient and public involvement techniques will support the research community in understanding the strengths and limitations of these approaches for application in their own work. In particular, understanding the participant experience will help enhance the future selection and use of such methods.

One vital area for understanding patient and public research priorities is low back pain (LBP), one of the most important causes of functional limitations and disability worldwide [9, 10]. Nearly a third of the US population over age 65 years experiences severe LBP, making it a leading reason for physician visits [11, 12]. A number of treatments are proposed as means to relieve LBP, ranging from physical therapy to surgical intervention, yet evidence on their effectiveness is inconsistent. While, in part, this relates to the relatively poor understanding of underlying mechanisms, the lack of high-quality research comparing options that mirror patient preferences for treatment is another cause [13, 14]. This disconnect between the information that people need to support decision-making and the available evidence is a recognized barrier and one that patient-centered outcomes research aims to address [3]. To expand evidence on effective treatments for LBP, the Back pain Outcomes using Longitudinal Data (BOLD) registry enrolled a group of older adults with LBP newly presenting to primary care to gather information about treatment pathways, healthcare utilization, and patient-reported outcomes [15]. In addition to generating knowledge about the clinical course of LBP, BOLD provides an opportunity to obtain input on research priorities from people with experience living with and navigating treatment decisions to maximize their health. Further, this informs ways that existing research infrastructures, such as patient registries, provide a mechanism for patient involvement in the prioritization of future research.

Developing infrastructure for and evidence on methods for involving stakeholders in the research process are important for guiding patient-centered outcomes research. In 2013, the Patient-Centered Outcomes Research Institute (PCORI) Methodology Committee published a recommendation to “support empirical research to assess and improve research prioritization methods for use by PCORI.” [16] Through a PCORI-funded study (Study of Methods for Assessing Research Topic Elicitation and pRioritization [SMARTER]), we sought to address this recommendation by comparing different methods for obtaining input on future research topics for LBP, with the aim of evaluating how prioritization methods (crowd-voting, focus groups using nominal group technique, and surveys using a modified Delphi method) compare across different domains regarding the ways participants rank research priorities and perceive their participation experience.

We hypothesized that the different techniques would produce similar rankings for the top five research priorities but differ in participant-rated experience, where greater participant interaction would receive better ratings.

## Methods

### Patient involvement in study design and conduct

**Fig. 1:**
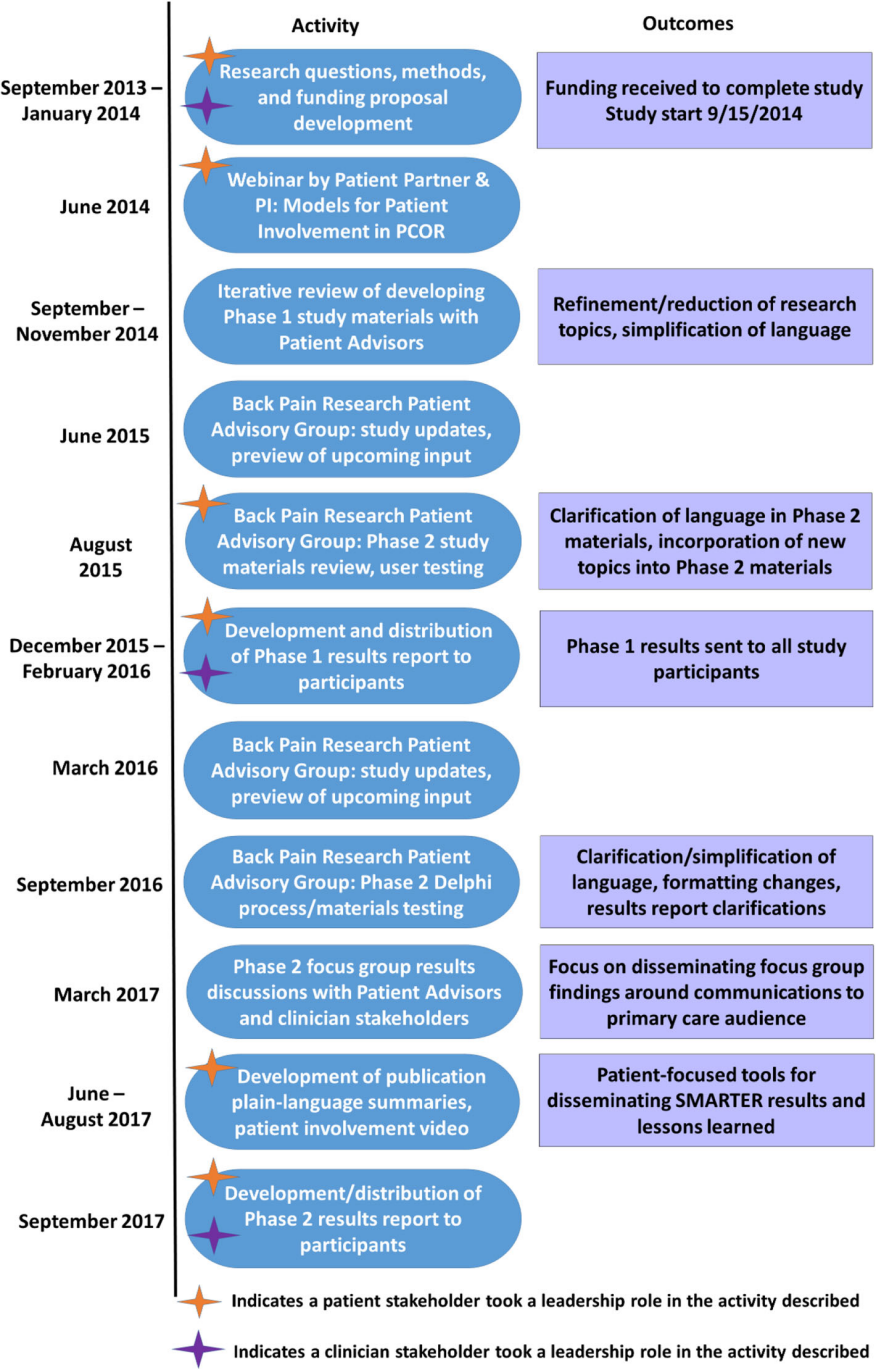
Patient and stakeholder engagement process in SMARTER

Patient and public involvement occurred throughout the project through both collaborative and consultative activities (Fig. 1) [17]. This included collaboration with a patient partner (MRS), who served as a member of the research team and participated in the development of the study from conception through study conduct; consultation with five patient advisors from the two BOLD clinical sites involved in recruiting participants for the registry (Henry Ford Health System [HFHS] in Michigan and Kaiser Permanente Northern California [KPNC]), which allowed for iterative consultation during study materials development; and finally, consultation with the CERTAIN Back Pain Research Patient Advisory Group, a committee of 10 patient advisors established in 2014 by researchers at the University of Washington (UW) to support patient involvement across a number of ongoing research initiatives [18]. Consultation with the CERTAIN Back Pain Research Patient Advisory Group throughout the study provided a forum for presenting and discussing project updates, obtaining input on study design decisions including evaluation, and discussing findings. Details about patient involvement in the design and conduct of the study have been published previously [19].

### Study design

This multi-phase project compared different quantitative and qualitative engagement methods for research [19]. In the study phase presented here, members of the BOLD registry were randomized in one of three prioritization activities (described below): crowd-voting [20], focus groups using nominal group technique, and a modified Delphi method using surveys [21, 22].

#### Online crowd-voting

Crowd-voting leverages online platforms to engage participants in discussion. This activity used IdeaScale, a secure, Internet-based community platform that allows participants to submit new ideas, vote on existing ideas, and interact with others through online, asynchronous discussion [20].

#### Focus groups with nominal group technique

Nominal group technique, a structured method for engaged problem-solving, is suitable for small group discussion on a defined topic, providing opportunity to generate new ideas. The method combines individual work and thought for new idea generation with moderated, interactive group discussion followed by prioritization of topics [21].

#### Modified Delphi method

In this study, the modified Delphi method used two rounds of mailed surveys to obtain input from respondents selected for experience in a given area. Participants reviewed a list of topics and provided individual ratings and commentary for each topic, as desired. During the second round, participants viewed their own responses in context of the group responses and additional commentary. Participants could adjust their ratings in the second round based on this new information [21].

Focus groups and the modified Delphi method are well-established consensus-building techniques, but they vary in level of participant interaction [21–23]. Crowd-voting provides efficiencies for obtaining input, with all participant interaction occurring online.

Activities used a prioritization list generated by BOLD participants in an earlier project phase [19, 24, 25]. Survey content was adapted from a list of 25 topics identified by primary care clinicians and researchers in 2009 and covered prevention, treatment options, diagnosis, communication, and outcomes of treatment [26]. Within-group work for each activity allowed for rating new topics generated by BOLD participants in the first phase of the study, as well as generation and prioritization of new topics during the activities [19, 25].

Institutional review boards at collaborating institutions (UW, HFHS, KPNC) approved the protocol for this study. All participants completed a consent form and a brief demographic form before the prioritization activity.

### Data management

UW served as the data coordination center for BOLD and subsequently this study, handling recruitment and follow-up of participants across the sites and providing a common infrastructure for management of study data. Demographic, evaluation, and Delphi survey data were entered into the secure and encrypted Research Electronic Data Capture (REDCap) software platform [27]. Audio recordings and transcriptions of focus group discussions and evaluation interviews were stored on a secure and encrypted system maintained at UW. Finally, crowd-voting data, captured on IdeaScale [20], a secure Internet-based community platform, were exported directly at the conclusion of the activity for analysis.

### Prioritization activity assignment and recruitment

BOLD participants were first asked to rank their choices of activities in order of preference. This was done to maintain a patient-centered approach to activity recruitment, as a slight preference for one activity might result in choosing to participate when asked. Each activity involved different levels of effort for participation. For example, focus groups require in-person attendance, whereas crowd-voting provides the opportunity to participate from home. Thus, when possible, we wanted to take preference into account.

To ensure equal representation from each study site, we stratified the sample by site. We then cross-tabulated the number who endorsed each activity to determine randomization probabilities. Participants from HFHS offered limited endorsement of crowd-voting and focus group activities. Across both sites, the mailed Delphi survey was the most highly endorsed, followed by focus groups, and then crowd-voting. We decided to randomize participants first to crowd-voting, then to focus groups, and finally to the Delphi survey. Not all eligible participants were assigned to an activity. When possible, group assignment probability was weighted by preference, subject to the constraints of the marginal total goals. For example, at HFHS, we were unable to take preference for crowd-voting or focus groups into account, given both the low number of participants at the site and the small number endorsing these activities. Those who preferred the Delphi survey were at most randomized with probability 0.8, second highest with probability 0.5, and third highest with probability 0.6. Because of the larger numbers at KPNC, we were able to include preference in the randomization for all activities at that site.

Participants were recruited approximately 6 weeks prior to the planned start of each activity. They were contacted by phone and provided a description of the activity, including the purpose, goals, expected time commitment, participant role, and incentives for participation. Recruitment continued until capacity was reached for each activity. We excluded participants from the crowd-voting activity if they did not have ready access to a computer or an active email address. Consented participants were contacted at 2 weeks and 2 days prior to an activity as a reminder of the event and to answer any questions.

### Priority-setting activities

Excluding descriptions of and instructions for each specific activity, all preparatory materials provided consistent messaging across all methods to reduce variation in external factors that could influence the experience or outcomes.

### Evaluation

To evaluate the quality of experiences and the perceived effectiveness of each activity, all participants received evaluation surveys (Additional file 1); a subset from each activity participated in semi-structured interviews selected to reflect a range of responses from evaluation surveys (Table 1). The evaluation survey assessed how effective each method was in meeting the overarching goals of PCOR as trustworthy, fair, balanced, respectful, and accountable [16, 28]. Questions allowed participants to evaluate the process and experience using a 10-point Likert-scale, where low numbers indicated low agreement with each item statement. We included open-ended questions to elicit input on aspects that participants liked most and least for each engagement activity. Crowd-voting and Delphi survey participants received the survey via Internet or mail, respectively, at the conclusion of the prioritization activity. Individuals participating in focus groups received the evaluation survey in person immediately following the activity.

**Table 1:** Evaluation interview guide questions

**Evaluation interview questions**	
1. **Tell me about your experience**	
• Why did you choose to participate in the (Activity) (e.g., why did you say yes, why did you follow-through with participation)?	
• What did you understand the purpose of the (Activity) to be?	
• To what extent did you interact with other participants in the (Activity)?	
2. **How could we improve (Activity) for others (e.g., clarity/readability of instructions, estimates of time spent, actual time commitment, technical support, communication with coordinators, etc.)?**	
3. **Prior to this activity you completed a survey mailed to you that asked you to rank individual low back pain research topics. Do you recall completing the survey?**	
• If no, skip	
• If yes, how would you compare the (Activity) to the first survey (e.g., ease of participating, understanding of what we were asking for, etc.)	
4. **In this project, we are trying to determine how patients can inform in the initial stages of medical research (i.e., identifying research topics and research priorities).**	
• How do you think researchers could do a better job of engaging patients in the initial stages of medical research?	
• What are other ways in which patients could contribute to medical research, beyond involvement as research study participants?	
• What role do you think patients should play in setting research priorities?	
• What effect do you think patients could have in being involved at this stage of the research process?	
5. **Do you have any other thoughts or comments you would like to share?**	

### Analytical and statistical approaches

To evaluate our hypothesis that participants would prefer methods with greater interaction, defined both as in-person interaction with other participants (focus groups greater than crowd-voting greater than Delphi survey), we analyzed the evaluation questions using descriptive statistics. We created categorical variables for experience quality based on the Likert Scale responses (0–4 = low, 5–7 = neutral, and 8–10 = high) to each item. We used the non-parametric Kruskal-Wallis test to compare the groups. A *p*-value < 0.05 indicated that at least one of the groups is different from the others.

We employed thematic analysis to document and examine patterns within and across the evaluation interview transcripts [29]. The interview transcripts were checked for accuracy, de-identified, and then uploaded into the web-based Dedoose qualitative analysis program [30]. The audio recordings from the evaluation interviews were transcribed. Two research team members (DL, SOL) read through transcripts and independently generated initial lists of codes related to descriptions of patient knowledge about the activity and perceptions of experience. The team members compared their code lists, explained code definitions, and reconciled their codes into one list. The final code list was presented and explained to the rest of the research team prior to being applied to the interview transcripts for engagement-related content. The coding team regularly communicated about the codes, code applications, and emerging themes.

We assessed differences in participant characteristics across the activities. Categorical data were compared using a Chi square test, except where noted, and continuous measures were compared using a t-test. Patient characteristics include geographic location, age group (65–74 years, 75–80 years, greater than 80 years), sex, education, marital status, disability as measured by the Roland Morris Disability Questionnaire (RMDQ), and duration of pain.

## Results

**Fig. 2:**
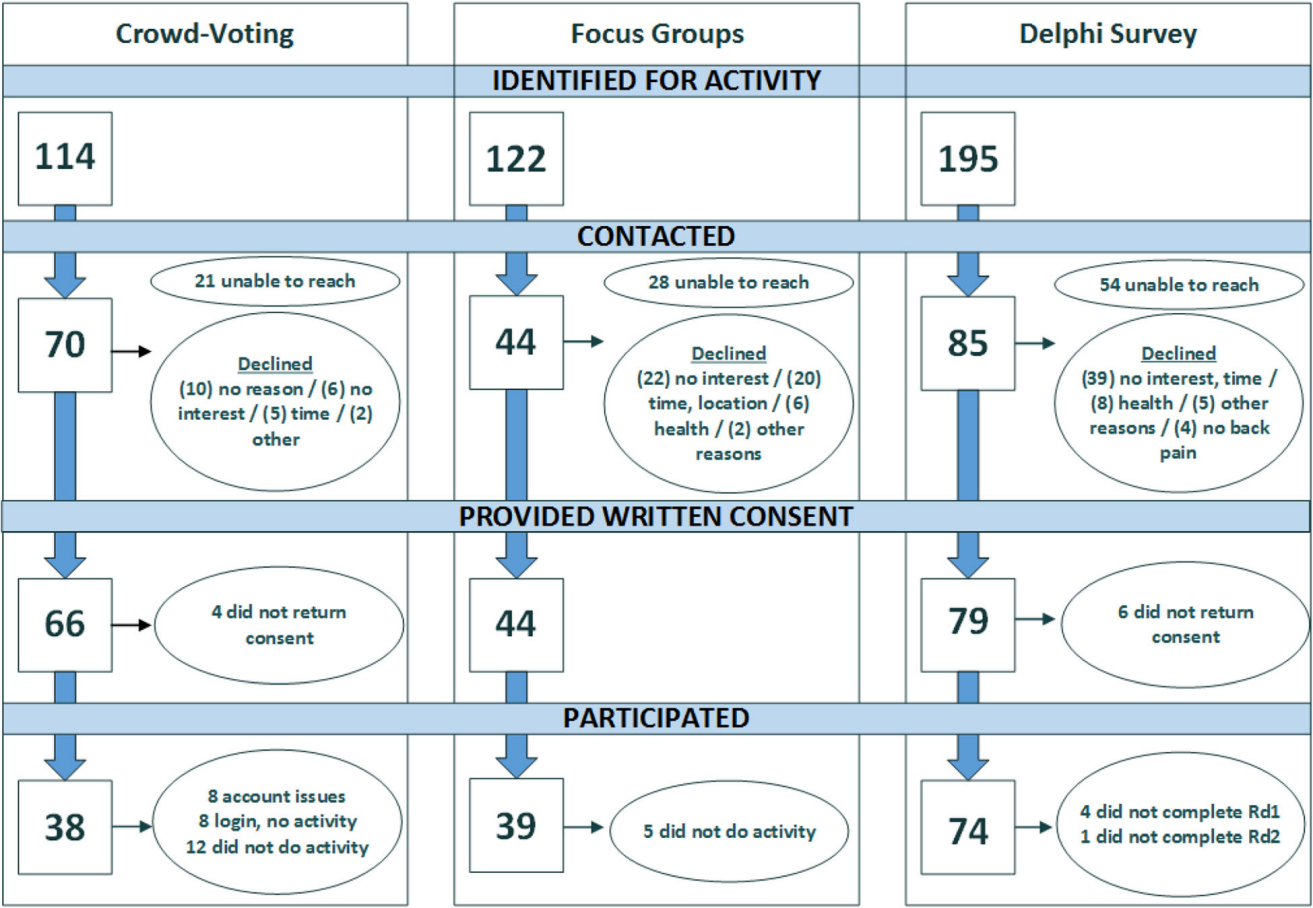
Phase 2 flow of participants through activities

Activities occurred from April 2016 through March 2017. The crowd-voting activity took place over a 6-week period. Six in-person focus groups were held, lasting approximately 3 h each. Participants in the Delphi survey completed surveys at their convenience within 2 weeks. There were 38 participants in the crowd-voting activity (54.3% participation), 39 participants in the focus group activity (88.6%), and 74 participants in the Delphi survey (87.1%) (Fig. 2). College educated participants were the majority in each activity, and there were more women than men in the focus groups and Delphi survey. Otherwise, demographics across the activities were similar (Table 2). Within the top five priorities, all groups prioritized “diagnosis-causes” and efforts related to treatment strategies (Table 3). Focus group and Delphi activities generated four priorities in common, crowd-voting and focus groups generated three, and crowd-voting and Delphi had just two priorities in common.

**Table 2:** SMARTER participant characteristics in each activity

Participant characteristics (n[%])	Crowd-voting	Focus group	Delphi survey	***p***-value
**N**	38	39	74	
**Age (mean [SD**^**a**^**])**^**b**^	74.03 (3.81)	75.14 (4.29)	77.19 (6.13)	0.03
**Sex**				
Female	18 (50%)	27 (71%)	44 (61%)	
Not reported	2	1	2	
**Race**^**c**^				0.18
Black or African American	2 (6%)	9 (26%)	8 (11%)	
Native American Indian or Alaska Native	1 (3%)	0 (0%)	0 (0%)	
Native Hawaiian or other Pacific Islander	1 (3%)	0 (0%)	0 (0%)	
Asian	2 (6%)	3 (9%)	2 (3%)	
White	30 (83%)	22 (65%)	60 (83%)	
Other	0 (0%)	0 (0%)	2 (3%)	
Multiple races	0 (0%)	0 (0%)	0 (0%)	
Not reported	2	5	2	
**Hispanic ethnicity**				0.45
Hispanic	0 (0%)	1 (3%)	4 (6%)	
Not reported	3	5	3	
**Education**^**d**^				0.04
Less than high school graduate	1 (1%)	0 (0%)	1 (1%)	
High school graduate or obtained general equivalent diploma	1 (3%)	4 (11%)	8 (11%)	
Associate’s degree	1 (3%)	3 ()	6 (8%)	
Some college	6 (16%)	8 (21%)	19 (26%)	
Four-year college graduate	10 (27%)	10 (26%)	15 (20%)	
Professional or graduate degree	19 (51%)	13 (34%)	25 (34%)	
Not reported	1	1	0	
**Marital status**^**e**^				0.77
Married	20 (54%)	17 (46%)	38 (51%)	
Living with partner	1 (3%)	3 (8%)	2 (3%)	
Divorced	6 (16%)	8 (22)	11 (15%)	
Separated	1 (3%)	0 (0%)	0 (0%)	
Never married	1 (3%)	1 (3%)	2 (3%)	
Widowed	8 (22%)	8 (22%)	21 (28%)	
Not reported	1	2	0	
**How long has low back pain been an ongoing problem for you?**^**f**^				0.11
3–6 months	1 (3%)	0 (0%)	1 (1%)	
6 months-1 year	0 (0%)	2 (5%)	2 (3%)	
1–5 years	9 (24%)	13 (34%)	30 (43%)	
More than 5 years	28 (74%)	23 (61%)	37 (53%)	
Not reported	0	1	4	
**How often has low back pain been an ongoing problem for you over the past 6 months?**^**g**^				0.60
Less than half the days in the past 6 months	21 (55%)	14 (37%)	34 (47%)	
At least half the days in the past 6 months	6 (16%)	10 (26%)	15 (21%)	
Every day or nearly every day in the past 6 months	11 (29%)	14 (37%)	23 (32%)	
Not reported	0	1	2	
**In the past 7 days, how would you rate your low back pain on average? (mean [SD])**	3.9 (1.82)	4.8 (2.33)	4.2 (2.13)	0.21

**Notes:** a) SD = standard deviation; b) 1 participant in the Delphi survey and 2 participants in the focus group activity were missing data on age; c) p-value for race determined from Chi-square test of white or Caucasian vs. any other race; d) p-value for education determined from Chi-square test of four-year college graduate or more vs. less than four-year college graduate; e) *p*-value for marital status determined from Chi-square test of married vs. any other status; f) *p*-value for “how long has low back pain been an ongoing problem for you?” determined from Chi-square test of more than 5 years vs. any other status; g) p-value for “how often has low back pain been an ongoing problem for you over the past 6 months?” determined from Chi-square test of all categories

**Table 3:** Prioritization activities and results

	Crowd-voting	Focus groups	Modified Delphi survey
Format	Online discussion via IdeaScale (secure, Internet-based community platform)	Moderated small-group discussion using nominal group technique (NGT)	Sequential mailed paper surveys
Participant role	Submit ideas and vote on ideas online	Generate new ideas and consensus through discussion	Provide written responses to exchange ideas and information
Type of interaction	Asynchronous	Balanced, in-person interactive	None
Staff role	Minimal group moderation to facilitate community involvement, including reminders and updates	Actively moderated group discussions	No direct participant interaction, compiled and mailed summary reports
Time frame (active involvement)	6 weeks	3 h	2 weeks
Compensation	US $25	US $100	US $50
Number of participants	38 of 100 invited	39 of 60 invited	74 of 90 invited
Top five priorities	1. Diagnosis – causes^a^ 2. Treatment – weight control & exercise^c^ 3. Treatment – physical health^b^ 4. Diagnosis – tests^c^ 5. Treatment – health system^a^	1. Treatment – health system^a^ 2. Diagnosis – causes^a^ 3. Communication – patient-provider communication^b^ 4. Treatment – self care^b^ 5. Treatment – physical health^b^	1. Treatment – self-care^b^ 2. Communication – patient-provider communication^b^ 3. Prevention – reduce disability^c^ 4. (tied) Treatment – health system^a^; Diagnosis – causes^a^

Note: a) found in all three activity groups; b) found in two groups; c) unique to a single group.

### Evaluation: survey responses

The evaluation survey was completed by 123 participants. Response rates to the evaluation survey were high for the Delphi (85.1%) and focus groups (87.2%) and moderate for crowd-voting (65.9%). Characteristics of evaluation survey participants are provided in Table 4.

**Table 4:** Characteristics of evaluation survey respondents

Participant characteristics (n[%])	Crowd-voting (***N*** = 26)	Focus group (***N*** = 34)	Delphi survey (***N*** = 63)	***p***-value
**Age**^**a**^**(mean [SD])**	74.36 (4.24)	75.19 (4.11)	76.79 (5.94)	0.03
**Sex**				0.65
Female	15 (60%)	23 (70%)	37 (61%)	
Not reported	0	1	2	
**Race**^**b**^				0.28
Black or African American	1 (4%)	7 (24%)	8 (13%)	
Native American Indian or Alaska Native	1 (4%)	0 (0%)	0 (0%)	
Native Hawaiian or other Pacific Islander	1 (4%)	0 (0%)	0 (0%)	
Asian	2 (8%)	2 (7%)	1 (2%)	
White	20 (80%)	20 (69%)	51 (84%)	
Other	0 (0%)	0 (0%)	1 (2%)	
Multiple races	0 (0%)	0 (0%)	0 (0%)	
Not reported	0	5	2	
**Hispanic ethnicity**				0.81
Hispanic	0 (0%)	1 (3%)	3 (5%)	
Not reported	2	4	3	
**Education**^**c**^				0.30
Less than high school graduate	0 (0%)	0 (0%)	1 (2%)	
High school graduate or obtained general equivalent diploma	1 (4%)	4 (12%)	7 (11%)	
Associate’s degree	0 (0%)	3 (9%)	6 (10%)	
Some college	6 (24%)	6 (18%)	15 (24%)	
Four-year college graduate	7 (28%)	9 (27%)	13 (21%)	
Professional or graduate degree	11 (44%)	11 (33%)	21 (33%)	
Not reported	0	1	0	
**Marital status**^**d**^				0.78
Married	14 (56%)	15 (47%)	33 (52%)	
Living with partner	0 (0%)	3 (9%)	2 (3%)	
Divorced	2 (8%)	7 (22%)	9 (14%)	
Separated	1 (4%)	0 (0%)	0 (0%)	
Never married	1 (4%)	1 (3%)	2 (3%)	
Widowed	7 (28%)	6 (19%)	17 (27%)	
Not reported	0	2	0	
**How long has low back pain been an ongoing problem for you?**^**e**^				0.27
3–6 months	0 (0%)	0 (0%)	1 (2%)	
6 months-1 year	0 (0%)	1 (3%)	2 (3%)	
1–5 years	7 (28%)	10 (30%)	24 (40%)	
More than 5 years	18 (72%)	22 (67%)	33 (55%)	
Not reported	0	1	3	
**How often has low back pain been an ongoing problem for you over the past 6 months?**^**f**^				0.34
Less than half the days in the past 6 months	15 (60%)	12 (36%)	33 (53%)	
At least half the days in the past 6 months	3 (12%)	10 (30%)	12 (19%)	
Every day or nearly every day in the past 6 months	7 (28%)	11 (33%)	17 (27%)	
Not reported	0	1	1	
**In the past 7 days how would you rate your low back pain on average? (mean [SD])**	3.9 (1.82)	4.9 (2.31)	4.1 (2.16)	0.15

**Note:**^a^) *p*-value determined from Kruskal-Wallis test; ^b^) p-value for race determined from Chi-square test of White or Caucasian vs. any other race; ^c^) *p*-value for education determined from Chi-square test of four-year college graduate or more vs. less than four-year college graduate; ^d^) *p*-value for marital status determined from Chi-square test of married vs. any other status; ^e^) p-value for “how long has low back pain been an ongoing problem for you?” determined from Chi-square test of more than 5 years vs. any other status; ^f^) *p*-value for “how often has low back pain been an ongoing problem for you over the past 6 months?” determined from Chi-square test of all categories

Focus groups received the most favorable ratings across dimensions of experience and process (Table 5). Crowd-voting received the lowest scores on process and experience, whereas the Delphi survey earned the lowest scores for eliciting questions and allowing feedback from activity coordinators. Participants across all activities reported high agreement with the importance of patient involvement in the identification and prioritization of research topics, and the majority of participants (92% crowd-voting, 91% focus groups, 92.1% Delphi survey) were willing to participate in future such studies.

**Table 5:** SMARTER evaluation survey responses^a^

	Crowd-voting	Focus groups	Delphi survey	***p***-value
**N**	26	34	63	
**I understood the tasks I was asked to perform as part of the activity**				**0.03**
0–4 (low)	2	0	0	
5–7 (neutral)	5	1	4	
8–10 (high)	19	33	59	
**I was able to ask questions and get feedback from the activity coordinators**				**0.006**
0–4 (low)	3	0	10	
5–7 (neutral)	3	0	14	
8–10 (high)	20	34	26	
**I felt different points of view were represented and shared**				**0.03**
0–4 (low)	3	0	2	
5–7 (neutral)	3	1	12	
8–10 (high)	19	33	48	
**Opportunities were provided to share opinions that differed from others in the group**				**0.01**
0–4 (low)	3	0	4	
5–7 (neutral)	3	0	9	
8–10 (high)	20	34	46	
**I feel the input I provided was valued**				**0.08**
0–4 (low)	4	0	2	
5–7 (neutral)	2	2	11	
8–10 (high)	20	31	50	
**My time was well spent on the activity**				**0.0002**
0–4 (low)	3	0	2	
5–7 (neutral)	6	1	8	
8–10 (high)	17	32	53	
**I was satisfied with my participation in the activity**				**0.0001**
0–4 (low)	6	0	1	
5–7 (neutral)	6	3	9	
8–10 (high)	14	30	53	
**I feel patients should have the opportunity to identify research topics for research**				**0.03**
0–4 (low)	0	0	6	
5–7 (neutral)	3	1	9	
8–10 (high)	23	31	47	
**I feel patients should have the opportunity to prioritize research topics for research**				**0.002**
0–4 (low)	1	0	7	
5–7 (neutral)	2	3	10	
8–10 (high)	23	28	45	
**Would you agree to participate in future studies to identify & prioritize research topics?**^**b**^				**0.08**
Yes	24	31	58	
No	2	0	4	

**Note:** Responses to all evaluation survey questions were not mandatory. Missing responses were excluded from analyses. ^a^) *p*-value determined from Kruskal-Wallis where a *p*-value <.05 indicates that at least one of the groups is different from the others; ^b^) *p*-value determined from Fisher’s Exact Test

The great majority of evaluation survey respondents provided open-ended responses to what they liked most about the activity (96.2% crowd-voting, 96.9% focus groups, 98.4% Delphi survey). The most frequently mentioned aspects were the opportunity to share ideas and hear/read about others’ experiences, the opportunity to contribute to research, and the focus on involving patients in the process. Focus group comments reflected enjoyment of in-person interaction; comments from crowd-voting and Delphi participants indicated a similar appreciation for learning from others, but the phrasing reflected the lower interaction among participants.

Overall, fewer participants commented on aspects of the activity they liked least (73.1% crowd-voting, 42.9% focus groups, 50.8% Delphi survey). The use of IdeaScale was unique to crowd-voting, and comments reflected challenges with the technology — both regarding the logistics for logging in as well as the user interface. Participants also noted that too many reminder and instructional emails with redundant content were sent over the course of the activity. Two respondents found the interaction experience as seeming isolated and impersonal. Comments from focus group participants centered primarily on logistical issues such as room temperature, distracting noise, and distance traveled to participate. Delphi participants noted the challenge of tracking their own participation, a circumstance that resulted from the delay between survey rounds. Three comments unique to Delphi respondents reflected a concern that their input was not relevant because their LBP was minimal.

Overall, the majority of participants commented favorably on their overall experience. This included enjoyment of participation, the focus of the research, and the opportunity it provided for patients. Recommendations for improvement often reflected the least liked aspects of the activities (e.g., improving the technology interface in crowd-voting). Cross-cutting recommendations for improving the activity focused on simplifying instructions and language as well as placing the study in a broader context. Crowd-voting and Delphi survey participants recommended including more information about the participants themselves, such as their diagnosis and duration of LBP in context with their responses.

### Evaluation: interview responses

Of those who responded to the evaluation survey, 31 participants completed interviews (11 crowd-voting, 10 focus group, 10 Delphi participants). Interviews lasted approximately 30 min. Interviews highlighted motivation for participating, understanding of the activity goals, perceptions of the activity process and experience, and views on the role patients should play in research. Table 6 presents representative open-ended comments. More detailed interview data by theme, discussed and stratified by activity participation, is available in Additional file 2.

**Table 6:** Representative quotes from interview participants

Motivation for participating in research activities	
• The reason why, just simply because somebody asked me, and why not? (participant, crowd-voting) • Yeah. I needed some information about back pain and back pain relief. And I saw different kind of inputs from participants and also from the people conducting the poll and the investigation. (participant, crowd-voting) • Well, I think research is always good. I mean, I’m at the other side of the spectrum in my life, but it might help other people as they approach their 50’s and so forth that maybe there’s things they can do while they’re still younger to be a preventative thing. (participant, Delphi) • Well, I thought I might learn something as well as if I had the opportunity maybe to contribute some of my experience from having a lot of low back problems. (participant, focus group)	
**Understanding of activity goals**	
• Well, it’s the idea, and we did this in the Forest Service, as well, when you had, from our research bureau, was getting research out to the -- to a broader audience, now how do you do that? You know, and one of the things is to direct the researcher to pick topics that are going to direct you toward a certain goal. (participant, crowd-voting) • In terms of where the emphasis should be, but so many of the questions had ... I mean, they were appropriate questions and appropriate priorities. Yeah, it was clear enough. (participant, Delphi) • Well, as I understood, it was to identify research topics which ordinarily would be identified without the kind of participation that this particular study included. (participant, crowd-voting) • It seemed to me that they were trying to decide where to focus their study. Because we were asked questions that, you know, gave opinions as to, you know, what should be followed up on, what type of information. (participant, focus group) • I understood the focus to be that you would collect information from patients, a large number of patients, and would assemble therefrom information that would be helpful to the medical profession in training new people or giving feedback on what might better help their patients. (participant, focus group)	
**Process measures**	
* Logistics*	• The electronic thing appealed, but, again, I, I didn’t get, I didn’t get the feedback. I don’t know if that’s the right word. The user interface satisfaction, I guess is what I’m going to say, that I would anticipate on doing things electronically rather than hardcopy. It just didn’t have that interaction that I was anticipating. (participant, crowd-voting) • So, you know, it didn’t take up a lot of time. The parking was adequate because it was right across the street from the facility. So, yes, I enjoyed participating. (participant, focus group) • I was surprised, correct me if I’m wrong, I almost had the feeling that you asked the same question several times. Am I correct about that? (participant, Delphi)
* Clarity of instructions & materials*	• I think, I think you did a really good job because like I don’t, I don’t, I’m a complete dinosaur and I don’t have any you know, I don’t have anything with push buttons, I don’t have a computer and so you got to me and without all the modern conveniences cause you talk to me on thephone and you sent me a letter with really easy instructions. (participant, focus group) • The way the questions were set up. Maybe if the instructions had been clearer, then the process would have been easier. (participant, crowd-voting) • I’ve been in the medical business, and I’m a teacher. I’m a toxicologist. I’m used to medical jargon and stuff. I wonder how many of the other participants were, but it is difficult to try to get the all-knowing, all-reaching question out there that’s understood. (participant, Delphi)
* Representation*	• Maybe we should cut down some selection. You don’t need do it if you rarely have back pain. Or you did have back pain and you don’t have back pain anymore. Or something like that. (participant, crowd-voting) • I also questioned why I was really a part of the survey. I was asked, so, therefore, this kind of thing I have no problem doing. My back issues are minimal. I’m sure you’re dealing with people for whom it’s a life changing event. Mine is not. (participant, focus group)
* Transparency & accountability*	• I think the online activity, because it was just like -- I mean, you could switch the comments on there, and you could hear other people’s comments or read other people’s comments, too. So I really liked the online survey. I liked that one. (participant, crowd-voting) • That’s really important, and you’ve done that, though. I’ve read responses that you sent me about three, 4 months ago, and I found them interesting. You know what, I even found it really interesting that one of my responses was in it. (participant, Delphi)
**Role of patients in research**	
* Importance*	• A lot of surveys, I participate in a lot of surveys and activities that provide feedback and information because I think it’s critical to get it from, shall we say, the horse’s mouth instead of guessing for researchers. (participant, crowd-voting) • Yeah, because, like, it’s like we’re the ones that, we’re the ones that are being, you know, being worked on, and so we should have some kind of say in what you’re working on. (participant, focus group) • That’s one of the reasons why I can respond so strongly to this is because to me it’s stupid to spend a lot of money and never ask the people that it’s going to affect. (participant, Delphi)
* Tasks*	• Well, they could, they ultimately could be one of the deciding factors as to whether something is funded or not. It’s all eventually about whether you get funded. (participant, crowd-voting) • By involving them as much as possible, even in framing the questions and the focus of the study. As I said, I think a lot of times research leaves out the very people that are most affected by the results of it. Anytime that they can be included from the very beginning of what’s even going to be studied is important, and that along the way as well. (participant, Delphi) • Maybe all patients should be given some kind of survey based on their level of injury. About their experiences with this and maybe things that they did that helped alleviate the pain besides what the doctor said. I don’t know if that’s a good answer, but that’s all I can come up with. (participant, focus group)

#### Motivation for participation

Interviewees voiced common motivations for participating, including interest in learning how to manage LBP, a desire to advance research that helps others, and the appeal of engaging in an activity focused on the patient perspective. The most frequent reason cited for participation was to learn more about LBP treatment, including both learning how other individuals manage their condition and learning if new approaches were available. Similarly, participants were interested in sharing their individual experiences to help others, and many interviewees noted that simply being asked motivated their participation. Finally, a few participants credited the emphasis on hearing from patients as motivation to participate.

#### Understanding of activity goals

Among the participants interviewed, the majority identified the purpose of the activity as understanding the patient experience of LBP, including treatments used, those found effective, and information needs about the condition. This perception was most pronounced among those who participated in the focus group activity. The minority of interviewees understood our goals to be identification and prioritization of research questions in LBP. Seven participants said the formal goal of the activity was unclear. Of note, across activities, participants in crowd-voting most often recognized the goal of prioritization compared with individuals in the Delphi survey and focus groups.

#### Process measures

All interviewees commented on process outcomes related to participation. Themes identified related to logistics, clarity of instructions and materials, representation of participants, and transparency/accountability.

##### Logistics

Comments regarding logistics for participation reflected the nuanced approach to each activity. Among participants in the crowd-voting activity, comments largely focused on technology. The concept of online participation appealed to a number of interviewees. However, technical issues were often cited as hindering participation (i.e., account set-up and log-in), as well as the user-interface for voting. Focus group participants commented favorably on the logistics and organization of the activity; however, some remarked that additional time for discussion would have been ideal.

Participants in the crowd-voting activity noted that more hands-on moderation to guide discussion among participants and provide structure to the activity would have been beneficial. Focus group participants remarked favorably on the group discussion and interaction; however, a couple of interviewees offered negative remarks about the final summarization of topics, suggesting the wrap-up devalued the discussion.

##### Clarity of instructions and materials

The clarity of instructions for participation and materials prompted mixed reviews. While some participants commented that activity instructions were straightforward, others found instructions lacked clarity and recommended simplification of language and steps. Participants with experience in the medical field or survey research acknowledged the difficulty of conveying complicated topics and crafting survey questions that adequately captured the breadth of information desired. Interviewees also commented on the research topics. A number noted that a lack of specificity among the topics made it challenging to tease out the importance of any one question over another.

##### Representation

A few individuals interviewed noted that their LBP was either minimal or resolved, and they were concerned that this diminished the value of their contributions in comparison to others who described greater disability or limitations resulting from significant LBP.

##### Transparency and accountability

A number of interviewees expressed interest in seeing activity results and understanding how results would be used by the medical research community.

#### Role of patients in research

Interviewees noted the importance of patient involvement in research, commenting on the ability of patients to provide researchers with insight into what is relevant and important for individuals living with LBP. By involving patients, it was felt, researchers can better understand how the condition affects a person’s life, subsequently making it more likely such evidence will be used. One participant noted the ethics of patient involvement, tying the importance of involving those who are the recipients of healthcare to the decisions about what to study.

Only a few interviewees articulated specific roles for patients, such as participating in focus group discussions and paper-based surveys. Beyond providing insight on experience, additional roles for patients in research included identifying research topics, refining research questions, and framing study design to ensure that the evidence generated would be relevant and actionable for patients. One person noted involvement in determining funding decisions for research.

## Discussion

Our study offers important insights into how interactive methods for involving patients and the public in patient-centered outcomes research compare. While the different methods yielded similar priorities for LBP, they differed in both how topics were ranked and in participant perceptions of experience.

### Results in context

Our findings suggest participants differ in their perceptions of experience and their understanding of the activity in which they took part. For the participants in the focus groups, interactions — with other participants and with the research team — appear to have the greatest influence on their perceptions. This aligns with our hypothesis. However, the misunderstanding among focus group participants concerning the purpose of the activity suggests that the input provided did not support the goal of generating research prioritization. We believe the differences in understanding could reflect the processes of the different activities. The attention placed on group interaction and discussion in the focus groups compared with the focus on ranking topics in the conduct of the Delphi survey and crowd-voting activities could influence participant understanding of the activity. Researchers should prioritize maximizing interaction among and between participants while also ensuring participants fully understand the goals and anticipated outcomes throughout the activity.

All activities allowed participants to provide commentary and generate new topics. Interaction among participants provided important insight and context regarding priorities. In focus group discussions, participants connected the topics to their personal experiences, revealing preferences and disinterest for particular topics at a level of detail that prioritization lists alone would not capture. Further, discussions highlighted how influences from external factors such as family or peer experience, recommendations from healthcare providers, or healthcare experiences inform views and perceptions. The value of this information, while difficult to quantify, is important for funders and researchers to consider when selecting methods for engagement.

### Results uptake

This project is timely, as national and international efforts to involve patients and the public in generating research portfolios grow. Understanding the effectiveness of different engagement activities will allow research teams to be more intentional regarding the approaches used in selecting methods to elicit input on research prioritization. Further, understanding participant experiences highlights areas for improving the use of these methods in the future. In our experience, working with people enrolled in a research registry provided an opportunity to hear from a community of individuals with direct experience in a defined area of health. By working with two registry sites, we reached a diverse group of individuals. Building upon the existing BOLD infrastructure allowed us to further cultivate an already-established relationship between the site teams and registry participants to conduct outreach. Staff contacted participants regularly over 2 years, establishing both a relationship and a bond of trust; these are both principles identified for meaningful patient engagement. This proved important for outreach. The majority of participants required more than one contact attempt, and we noted that follow-up calls often resulted in participation. Further efforts to expand engagement within patient registries should focus on techniques that support involvement, considering the resources necessary for conducting such work.

Another interesting finding pertains to participant views of representation. Patient-centered outcomes research encourages representation from a range of perspectives and experiences with a condition. In our study, participants noted concern about the value of their contributions when they experienced minimal back pain compared with others who experienced greater pain and disability as a result of the condition. Further work should focus on patient and public perception of appropriate representation in research.

More work is needed to build understanding around the important role that patients play to define, guide, and support research. Participants in this study expressed their belief in the value of patient involvement as a means to ensure the relevance and importance of the research conducted. While this supports the goal of patient-centered outcomes research, the roles articulated for patients were frequently limited to consented participation in research studies. This demonstrates that the concept of patient and public involvement in research is still new and not widely normalized in non-academic, non-research communities. Lack of understanding about the opportunity for patients and members of the public to inform and collaborate on research study teams may also reflect the lack of understanding of the primary goal across prioritization activities. Our team took this into account when planning for dissemination of study results and developed a video in conjunction with the CERTAIN Back Pain Research Patient Advisory Group to better explain and illustrate the role patients can play in research (Fig. 1). However, more work is needed to communicate the role that patients play as partners on research teams in order to continue building knowledge about and capacity for patient involvement in future research.

### Study limitations

Our study focused on assessing prioritization methods among older adults with LBP and thus may not be generalizable for other health conditions or populations. For example, rare conditions may require methods that allow remote participation if it is not feasible to convene people in a central location due to geographic diversity [31, 32] or limitations imposed by certain health conditions. For example, face-to-face contact between people living with cystic fibrosis is not advised due to the risk of cross-infection [33]. In such circumstances, online engagement is warranted to support involvement. Characteristics of the population may also inform preferences for engagement methods. The BOLD population is limited to those with LBP 65 years and older. Thus, findings may not be generalizable to younger patient populations for whom technology is more ingrained in day-to-day communication and activities. Our experience with the BOLD registry indicated that online interaction would not be a preferred mode of communication from the outset, which informed our decision to use mailed surveys in place of online completion of the prioritization questionnaire.

We experienced technological challenges with the crowd-voting platform (IdeaScale) that resulted in lower participation rates.

Participants in our study were predominantly white, reported higher levels of formal education, and represented people enrolled in a research registry. Thus, it is important to recognize that the views and perspectives of participants are not generalizable to all people with LBP. While registries, by nature of bringing together people with a common health experience, offer a unique opportunity for involvement in identifying and prioritizing research, it is important to understand the underlying characteristics of participants and what views are underrepresented or missing.

Our team was concerned that conventional randomization would preclude participation (and limit successful study conduct) based on patient factors such as ability to travel and comfort with technology. To address this, we asked participant preference for activity participation. To control for bias introduced with this approach, we intended to use randomization by minimization to allocate participants to interactive prioritization activities while ensuring balance across groups on important factors that could heavily influence the ranking of priorities. Due to limited preference among participants for crowd-voting (*n* = 21), we had to modify our plan. In this case, preference for a given approach might reflect underlying participant characteristics associated with how topics are prioritized. This is a noted limitation of this study.

Obtaining input for research via survey outreach through a patient registry is more appropriate to supplement or complement collaborative research endeavors that provide dialogue and co-production of research reflective of partnership. It is important to recognize that providing input on future research topics is a different task than consenting to participate in a research study. Requesting involvement in other research activities outside of BOLD was not part of initial consent or discussions regarding participation. This could have resulted in a lack of understanding about how this new activity fit within the purpose of BOLD, thus limiting participation.

Our study highlights several areas for future research. Comparison of research prioritization methods among different health conditions (e.g., acute, rare, and/or co-existing, etc.) as well as comparison of research prioritization methods among diverse and underrepresented populations will help inform how best to further tailor approaches for patient and public involvement in research. Further exploration to compare research prioritization methods among different stakeholder groups, including multi-stakeholder groups, will inform nuances for how different methods perform amongst groups representing different perspectives, experiences, and backgrounds.

## Conclusions

Patient and public involvement in research prioritization supports the goal of patient-centered outcomes research to pursue timely and important healthcare questions. Our study demonstrates that the diverse methods used in research prioritization yield similar priorities but differ in both the ways that priorities are ranked and in participant experience. Further, activities with higher levels of interaction among participants and with the research team yield more satisfaction but may require extra focus to successfully convey the core purpose of the study.

## Supplementary information

**Additional file 1.** Appendix 1: Phase 2 evaluation surveys

**Additional file 2.** Appendix 2: Selected evaluation interview quotes GRIPP2

## Data Availability

The datasets generated and/or analyzed during the current study are not publicly available, as study participants were not informed that study data would be made available to the public. Data are available from the corresponding author on reasonable request.

## Abbreviations

BOLD: Back pain Outcomes using Longitudinal Data registry
HFHS: Henry Ford Health System
KPNC: Kaiser Permanente Northern California
LBP: Low back pain
NGT: Nominal group technique
PCOR: Patient-centered outcomes research
PCORI: Patient-Centered Outcomes Research Institute
REDCap: Research Electronic Data Capture
RMDQ: Roland Morris Disability Questionnaire
SMARTER: Study of Methods for Assessing Research Topic Elicitation and pRioritization
UW: University of Washington
