# Supplementary material for: Comparing three approaches for involving patients in research prioritization: a qualitative study of participant experiences
Source: Res Involv Engagem. 2020 May 1;6:18. doi: 10.1186/s40900-020-00196-4 (PMC7195769; doi:10.1186/s40900-020-00196-4)
Supplement: Supplementary file 1 — Additional file 1. Appendix 1: Phase 2 evaluation surveys [file 40900_2020_196_MOESM1_ESM.pdf]

Appendix 1. Phase 2 Evaluation Surveys

Crowd-voting evaluation survey

Focus groups evaluation survey

Delphi survey evaluation survey

Evaluating Your Experience

Following are some statements that you might make about your recent involvement in prioritizing low back pain research topics. Please circle the one number on each scale that best describes your overall experience. There are no right or wrong answers. We are only interested in how you feel about your experience.

1. The materials provided prior to the activity were helpful for my participation. *(please circle one number)*

**NOT AT ALL**	**0**	**1**	**2**	**3**	**4**	**5**	**6**	**7**	**8**	**9**	**10**	**COMPLETELY**

2. I understood the goal for the activity I participated in. *(please circle one number)*

**NOT AT ALL**	**0**	**1**	**2**	**3**	**4**	**5**	**6**	**7**	**8**	**9**	**10**	**COMPLETELY**

3. I understood the tasks we were asked to perform as part of this activity. *(please circle one number)*

**NOT AT ALL**	**0**	**1**	**2**	**3**	**4**	**5**	**6**	**7**	**8**	**9**	**10**	**COMPLETELY**

4. I had a clear understanding of the process used to rank research topics. *(please circle one number)*

**NOT AT ALL**	**0**	**1**	**2**	**3**	**4**	**5**	**6**	**7**	**8**	**9**	**10**	**COMPLETELY**

5. I was able to ask questions and get feedback from the activity coordinators. *(please circle one number)*

**NOT AT ALL**	**0**	**1**	**2**	**3**	**4**	**5**	**6**	**7**	**8**	**9**	**10**	**VERY MUCH**

6. I felt different points of view were represented and shared. *(please circle one number)*

**NOT AT ALL**	**0**	**1**	**2**	**3**	**4**	**5**	**6**	**7**	**8**	**9**	**10**	**COMPLETELY**

7. Opportunities were provided to share opinions that differed from others in the group. *(please circle one number)*

**NOT AT ALL**	**0**	**1**	**2**	**3**	**4**	**5**	**6**	**7**	**8**	**9**	**10**	**VERY MUCH**

8. I feel the input I provided was valued. *(please circle one number)*

**NOT AT ALL**	**0**	**1**	**2**	**3**	**4**	**5**	**6**	**7**	**8**	**9**	**10**	**VERY MUCH**

9. My time was well spent on this activity. *(please circle one number)*

**NOT AT ALL**	**0**	**1**	**2**	**3**	**4**	**5**	**6**	**7**	**8**	**9**	**10**	**VERY MUCH**

10. I was satisfied with my participation in this activity. *(please circle one number)*

**NOT AT ALL**	**0**	**1**	**2**	**3**	**4**	**5**	**6**	**7**	**8**	**9**	**10**	**VERY MUCH**

11. I was satisfied with the final list of research priorities generated through this activity. *(please circle one number)*

**NOT AT ALL**	**0**	**1**	**2**	**3**	**4**	**5**	**6**	**7**	**8**	**9**	**10**	**VERY MUCH**

12. Would you agree to participate in future studies for identifying and prioritizing research topics? *(please check one box)* ☐ Yes ☐ No

13. What did you like MOST about participating in this activity?

14. What did you like LEAST about participating in this activity?

15. How could this activity have been improved?

For focus group and online crowd-voting participants only:

16. *I had an opportunity to learn from others' experience and points of view* (*please circle one number)*

NOT AT ALL	0	1	2	3	4	5	6	7	8	9	10	VERY MUCH

17. The focus group facilitator was effective in managing the group discussion. *(please circle one number)*

NOT AT ALL	0	1	2	3	4	5	6	7	8	9	10	VERY MUCH

18. Conflicts and disagreements among participants were well managed *(please circle one number):*

☐ Not applicable

NOT AT ALL	0	1	2	3	4	5	6	7	8	9	10	COMPLETELY

We would like to follow-up with 10 people from each of the 3 activities to hear more about your experience and how the activities could be improved. We will randomly choose 10 people to contact from each activity to invite you to talk with us for up to an hour. If you do NOT want to be contacted for this additional interview, please check this box: ☐ Please do NOT contact me.

Evaluating Your Experience

The following are some statements that you might make about your recent involvement in a focus group to prioritize low back pain research topics. Please circle one number on each scale that best describes your overall experience. There are no right or wrong answers. We are only interested in how you feel about your experience.

1. The materials given to me before the focus group were helpful for my participation. *(please circle one number)*

**NOT AT ALL**	**0**	**1**	**2**	**3**	**4**	**5**	**6**	**7**	**8**	**9**	**10**	**COMPLETELY**

2. I understood the goal for the focus group I participated in. *(please circle one number)*

**NOT AT ALL**	**0**	**1**	**2**	**3**	**4**	**5**	**6**	**7**	**8**	**9**	**10**	**COMPLETELY**

3. I understood the tasks I was asked to perform as part of the focus group. *(please circle one number)*

**NOT AT ALL**	**0**	**1**	**2**	**3**	**4**	**5**	**6**	**7**	**8**	**9**	**10**	**COMPLETELY**

4. I was able to ask questions and get feedback from the activity coordinators. *(please circle one number)*

**NOT AT ALL**	**0**	**1**	**2**	**3**	**4**	**5**	**6**	**7**	**8**	**9**	**10**	**VERY MUCH**

5. I felt different points of view were represented and shared. *(please circle one number)*

**NOT AT ALL**	**0**	**1**	**2**	**3**	**4**	**5**	**6**	**7**	**8**	**9**	**10**	**COMPLETELY**

6. Opportunities were provided to share opinions that differed from others in the group. *(please circle one number)*

**NOT AT ALL**	**0**	**1**	**2**	**3**	**4**	**5**	**6**	**7**	**8**	**9**	**10**	**VERY MUCH**

7. I feel the input I provided was valued. *(please circle one number)*

**NOT AT ALL**	**0**	**1**	**2**	**3**	**4**	**5**	**6**	**7**	**8**	**9**	**10**	**VERY MUCH**

8. My time was well spent on the focus group. *(please circle one number)*

**NOT AT ALL**	**0**	**1**	**2**	**3**	**4**	**5**	**6**	**7**	**8**	**9**	**10**	**VERY MUCH**

9. I was satisfied with my participation in the focus group. *(please circle one number)*

**NOT AT ALL**	**0**	**1**	**2**	**3**	**4**	**5**	**6**	**7**	**8**	**9**	**10**	**VERY MUCH**

10. I was satisfied with the final list of research priorities generated through the focus group. *(please circle one number)*

**NOT AT ALL**	**0**	**1**	**2**	**3**	**4**	**5**	**6**	**7**	**8**	**9**	**10**	**VERY MUCH**

11. I had an opportunity to learn from others' experience and points of view (*please circle one number)*

**NOT AT ALL**	**0**	**1**	**2**	**3**	**4**	**5**	**6**	**7**	**8**	**9**	**10**	**VERY MUCH**

12. The focus group facilitator was effective in managing the group discussion. *(please circle one number)*

**NOT AT ALL**	**0**	**1**	**2**	**3**	**4**	**5**	**6**	**7**	**8**	**9**	**10**	**VERY MUCH**

13. Conflicts and disagreements among participants were well managed *(please circle one number):*

☐ Not applicable

**NOT AT ALL**	**0**	**1**	**2**	**3**	**4**	**5**	**6**	**7**	**8**	**9**	**10**	**COMPLETELY**

14. I feel patients should have the opportunity to **identify** research topics for researchers. (*please circle one number)*

**NOT AT ALL**	**0**	**1**	**2**	**3**	**4**	**5**	**6**	**7**	**8**	**9**	**10**	**VERY MUCH**

15. I feel patients should have the opportunity to **prioritize** research topics for researchers. (*please circle one number)*

**NOT AT ALL**	**0**	**1**	**2**	**3**	**4**	**5**	**6**	**7**	**8**	**9**	**10**	**VERY MUCH**

16. Would you agree to participate in future studies for identifying and prioritizing research topics? *(please check one box)* ☐ Yes ☐ No

17. What did you like MOST about participating in the focus group?

18. What did you like LEAST about participating in the focus group?

19. How could the focus group have been improved?

We would like to follow-up with 10 people to hear more about your experience and how the activities could be improved. We will randomly choose 10 people to talk with us over the phone about their experience, for up to 30 minutes. If you do NOT want to be contacted for this additional interview, please check this box: ☐ Please do NOT contact me.

Evaluating Your Experience

The following are some statements that you might make about your recent involvement in a mailed survey activity to prioritize low back pain research topics. Please circle one number on each scale that best describes your overall experience. There are no right or wrong answers. We are only interested in how you feel about your experience.

1. The materials given to me before the mailed survey activity were helpful for my participation. *(please circle one number)*

**NOT AT ALL**	**0**	**1**	**2**	**3**	**4**	**5**	**6**	**7**	**8**	**9**	**10**	**COMPLETELY**

2. I understood the goal for the mailed survey activity. *(please circle one number)*

**NOT AT ALL**	**0**	**1**	**2**	**3**	**4**	**5**	**6**	**7**	**8**	**9**	**10**	**COMPLETELY**

3. I understood the tasks I was asked to perform as part of the mailed survey activity. *(please circle one number)*

**NOT AT ALL**	**0**	**1**	**2**	**3**	**4**	**5**	**6**	**7**	**8**	**9**	**10**	**COMPLETELY**

4. I was able to ask questions and get feedback from the activity coordinators. *(please circle one number)*

**NOT AT ALL**	**0**	**1**	**2**	**3**	**4**	**5**	**6**	**7**	**8**	**9**	**10**	**VERY MUCH**

5. I felt different points of view were represented and shared. *(please circle one number)*

**NOT AT ALL**	**0**	**1**	**2**	**3**	**4**	**5**	**6**	**7**	**8**	**9**	**10**	**COMPLETELY**

6. Opportunities were provided to share opinions that differed from others in the group. *(please circle one number)*

**NOT AT ALL**	**0**	**1**	**2**	**3**	**4**	**5**	**6**	**7**	**8**	**9**	**10**	**VERY MUCH**

7. I feel the input I provided was valued. *(please circle one number)*

NOT AT ALL	0	1	2	3	4	5	6	7	8	9	10	VERY MUCH

8. My time was well spent on the mailed survey activity. *(please circle one number)*

NOT AT ALL	0	1	2	3	4	5	6	7	8	9	10	VERY MUCH

9. I was satisfied with my participation in the mailed survey activity. *(please circle one number)*

NOT AT ALL	0	1	2	3	4	5	6	7	8	9	10	VERY MUCH

10. I feel patients should have the opportunity to **identify** research topics for researchers. (*please circle one number)*

NOT AT ALL	0	1	2	3	4	5	6	7	8	9	10	VERY MUCH

11. I feel patients should have the opportunity to **prioritize** research topics for researchers. (*please circle one number)*

NOT AT ALL	0	1	2	3	4	5	6	7	8	9	10	VERY MUCH

12. Would you agree to participate in future studies for identifying and prioritizing research topics? *(please check one box)* ☐ Yes ☐ No

13. What did you like MOST about participating in this activity?

14. What did you like LEAST about participating in this activity?

15. How could this activity have been improved?

16. Is there anything else you would like to share about your experience?

We would like to follow-up with 10 people to hear more about your experience and how the activities could be improved. We will randomly choose 10 people to talk with us over the phone about their experience, for up to 30 minutes. If you do NOT want to be contacted for this additional interview, please check this box:

☐ Please do NOT contact me.
