# Supplementary material for: Comparing three approaches for involving patients in research prioritization: a qualitative study of participant experiences
Source: Res Involv Engagem. 2020 May 1;6:18. doi: 10.1186/s40900-020-00196-4 (PMC7195769; doi:10.1186/s40900-020-00196-4)
Supplement: Supplementary file 2 — Additional file 2. Appendix 2: Selected evaluation interview quotes GRIPP2 [file 40900_2020_196_MOESM2_ESM.pdf]

Appendix 2. Selected Evaluation Interview Quotes

Evaluation interview transcript data by theme discussed and activity participation.

Activity	Crowd-Voting	Focus Group	Delphi
**Motivation for participating in research activities**			
	- The reason why, just simply because somebody asked me, and why not?	- Well, it seemed like it was a good idea. To me, it seemed a very good idea to give feedback to the medical profession on what their patients thought was helpful. And my back pain is not so severe that it causes me irrational problems. So, I thought that maybe I could be of some help and it felt good to be there.	- Well, I think research is always good. I mean, I'm at the other side of the spectrum in my life, but it might help other people as they approach their 50's and so forth that maybe there's things they can do while they're still younger to be a preventative thing.
	- It was requested and I said, sure no problem. If it's anything it would help other people in the same situation, and wind up helping me too, why not?	- I've been, oh, having lower back problems for quite a few years and thought it might be interesting going to learn something new and how to correct it and see if I'm the only one having those kind of issues. And it was a very, very educational group.	- I think the reward for participating in the survey is learning more about what in general the best ways to deal with the back pain ... Treat it or reduce it or so forth. The reward is the information
	- Yeah. I needed some information about back pain and back pain relief. And I saw different kind of inputs from participants and also from the people conducting the poll and the investigation.	- Well, I think we need to do as much as we can to try to bring medical community and patients together to make their treatments more effective and to have intercommunication.	- It was kind of a new approach and that kind of intrigued me. I've never seen anybody dealing with back pain or most any pain in the fashion your questionnaire was going towards. That was intriguing and like a fish being hooked I just wanted to follow along and see where it came out.
	- Well it was something I could do and it was interesting. I'm not, I'm not like some of the people that participated who have back pain all the time. So it wasn't like something I had to urgently, but I do have it once in a while and several times I've seen my [institution name] doctor and he's sent all kinds of tests and other things to see if that was it. So I guess I wanted to know if there was	- Well, I thought I might learn something as well as if I had the opportunity maybe to contribute some of my experience from having a lot of low back problems.<br>- Well, I thought I could get some additional information. I have back pain. I have sciatica. In fact, I'm going through an episode right now. And I thought maybe getting with other people who had similar issues with pain, I could learn something from the group.	- The reason why I decided to participate is because of just the concept of asking the actual patients for their participation and their feedback. Oftentimes research doesn't include that important part of who's being effected. The fact that it was based on it was what attracted me, and then of course, when I started to do it - it was interesting, and I thought much more valuable than having outside

	anything different or new that I didn't know about because when it hurts, it really hurts, but as I said, it's not every day so it's different.		researchers who may or may not have ever had a back pain themselves deciding what to study.
**Understanding of activity goals**			
	- Well, it's the idea, and we did this in the Forest Service, as well, when you had, from our research bureau, was getting research out to the -- to a broader audience, now how do you do that? You know, and one of the things is to direct the researcher to pick topics that are going to direct you toward a certain goal.	- Well, I thought it was an opportunity to bring people together that were having similar problems with their back, you know. [Inaudible] pain and that -- and to find out how they felt about it, what they had experienced, what they were doing. You know, it -- it made you feel that you were not alone, that there were others that were experiencing the same thing.	- What it read to me was that you were interested in seeing what other information would be useful to people with bad backs, and what things could be done to ... That they wouldn't have to go out searching for additional help, that they could possibly manage the problem themselves.
	- I understood it to be sort of a method of prioritizing research topics and that was why I kind of used to kind of look up online and look up elsewhere to see how they could help me.	- I understood the focus to be that you would collect information from patients, a large number of patients, and would assemble therefrom information that would be helpful to the medical profession in training new people or giving feedback on what might better help their patients.	- In terms of where the emphasis should be, but so many of the questions had ... I mean, they were appropriate questions and appropriate priorities. Yeah, it was clear enough.
	- It was meant to go towards the research on giving the primary care person the right things to ask the patient. So that they could either help themselves or get help.	- I know it was to find out how many people suffer from lower back pain. And, what kinds of things that they were doing to help themselves, I think.	- Well, I think part of it was surveying people who had problems, but also getting their opinions as to what treatment's available, what they themselves could be doing that would be better, and also just to further the spectrum of information to put out there for the coming generations.
	- Well as I understood it was to identify research topics which ordinarily would be identifies without the kind of participation that this particular study included	- It seemed to me that they were trying to decide where to focus their study. Because we were asked questions that, you know, gave opinions as to, you know,	- Again, just trying to remind myself, this last bit of survey questions dealt with what topics we would like to explore, what topics need to be explored.

	- Well, what I understood is you guys were trying to get input about laymen people like us think about what can be done to help with the back problems and what causes back problems, and, you know, I guess they know everything about what causes them, but it does a lot of different things that come into view that I hadn't thought about before.	what should be followed up on, what type of information.	
**Process measures**			
Logistics	- As I said, my impression was positive about the whole thing. And the part [inaudible] I think we were in that website for, I think, for a month or something like. It was pretty long. And I was logging in pretty much every day and looking up and then also mentioned [inaudible] every once in a while.	- I enjoyed the focus group better. Because that was my interaction with others and I could see from things that they were saying how some of this applied to me more so than other things. So, I enjoyed the focus group more.	- On the negative side, there was some problem figuring out if I'd answered by mail or by computer. That got resolved, and that's the last I heard of it.
	- The electronic thing appealed but again, I, I didn't get, I didn't get the feedback. I don't know if that's the right word. The user interface satisfaction, I guess is what I'm going to say, that I would anticipate on doing things electronically rather than hardcopy. It, just didn't have that interaction that I was anticipating.	- So, you know, it didn't take up a lot of time. The parking was adequate because it was right across the street from the facility. So, yes, I enjoyed participating.	- I was surprised, correct me if I'm wrong, I almost had the feeling that you asked the same question several times. Am I correct about that?
	- I mean, it was pretty straight forward. There was a set up introductory kind of thing, and then a list of things to vote on, and a request to add items. And [inaudible]. And I stayed consistent	- Yeah, I mean I think, you know, when you finally started to, you know, when she was trying to sum it up I thought that was useless. Because I thought that the doctors would get more out of just the	- I wasn't sure about some of the questions. I would go back and read them and I would have my wife read them because I'm blind.

	kind of reminder to participate. And then, and then the close out.	array of comments that they have, that the people have.	
Clarity of instructions and materials	- Perhaps the clarity of the instructions, and perhaps a little bit more on the purpose. I don't know, I didn't get excited about it as a research topic. I didn't get excited about, gee I'm participating in something that's going to, going to result in, in an improvement in some process	- I think, I think you did a really good job because like I don't, I don't, I'm a complete dinosaur and I don't have any you know, I don't have anything with push buttons, I don't have a computer and so you got to me and without all the modern conveniences cause you talk to me on the phone and you sent me a letter with really easy instructions.	- As I said, I thought they were very easy. The directions were easy. The statements were all, the purpose, everything was very well stated. I was very impressed.
	- There was a lot of scattered information. It wasn't concise like in a book format, sort of like that. Probably it was good. I mean the discussion was positive.	- They were very informative. They may -- kept the group together, you know. They were the guidance, but they didn't take it over and they allowed for the participants to give their views about certain things. But yeah, they were very clear in their instructions as to what they wanted us to do.	- I've been in the medical business and I'm a teacher. I'm a toxicologist. I’m used to medical jargon and stuff. I wonder how many of the other participants were but it is difficult to try to get the all knowing, all reaching question out there that's understood.
	- The way the questions were set up. Maybe if the instructions had been clearer, then the process would have been easier.	- They could have worked a little better on the instructions. We just kind of ended up -- these cards we were not quite sure what to do with them. A little better explanation or maybe an example of what we should be doing with them.	- At this point I don't have the final results to see what's happened, but I thought the survey overall was fairly good. One thing I was a little bit, I guess, unsure of was that I thought when we originally signed on for it there was going to be a one or two-hour session where, as a questionnaire, it was going to be very extensive questions. And so I thought that as we finished it it was much more general than I had figured that the experience would be, but I was fine with it.
	- Maybe we should cut down some selection. You don't need do it if you rarely have back pain. Or you did have back pain and you don't	- I didn't find it difficult, and I guess I more or less enjoyed doing it. I enjoyed doing the survey. Because I don't have constant back pain, I might not have been as	- I thought I talked to someone, but indicated that my back pain had been resolved a year or two ago, and was concerned that my experience wasn't recent enough,

Representation	have back pain anymore. Or something like that.	informative as somebody who suffers with it all the time.	that you might not want the information. I was told that you were really looking for people that had participated, and if their problems had been corrected you wanted their information as well, so I decided to go ahead and join in.
	- You know and you're counting a huge group of people with different problems. I did read some of them things. Some of these people had much more problems than I do. I can actually -- at the moment I'm not suffering from back pain at all.	- I also questioned why I was really a part of the survey. I was asked so therefore this kind of thing I have no problem doing. My back issues are minimal. I'm sure you're dealing with people for whom it's a life changing event. Mine is not.	- I noticed that there were -- what -- six females and two males. I don't think it needs to be gender even at all, but I did feel like that the other male and I might have not represented the -- your audience pretty well. We might have been different from a large number of your audience. And, of course, you have many others -- I mean I mentioned you took care of that. Gender balance is sometimes important. It might also be interesting to have groups that are all one gender because sometimes our responses are different from the other genders present.
Transparency & Accountability	- It was good but I needed to see the final report and also the idea of -- they have discovered -- they have to tell the people with lower back pain. Actually I waited for -- well after that process was finished and I sent an e-mail to the organizers and then she responded to that they have sent the report out. I haven't seen it yet.	- I thought some good thoughts were presented by the group. And I guess curiosity side to myself and probably others in the room about how will that information gets dissected and what becomes of it at some point.	- That's really important, and you've done that though. I've read responses that you sent me about three, four months ago, and I found them interesting. You know what, I even found it really interesting that one of my responses was in it.
	- I think the online activity, because it was just like -- I mean, you could switch the comments on there, and you could hear other people's	- Well I really think the people that are the ones that are hurting that are ones that are going to get the proper answers. I don't know if I'm going to give you a	

	comments or read other people's comments, too. So I really liked the online survey. I liked that one.	proper answer right now but I believe this is the only way that you get the true feeling of people and exactly what's going on, the pain they have or whatever they don't have when you're doing stuff. I just happen to think that again it goes back to are they really interested? Are they really listening? And if they don't like what they hear are they just going to sluff that off and just take what they like to hear from certain people, you know, I mean from what they want to hear.	
**Role of patients in research**			
Importance	- Well actually I think it would be very useful. I'm not even sure how you would do research without having patient involvement.	- I mean I think your goal is good because I'll bet there's a lot of wasted time in labs. Trying to find out things that aren't necessarily imminent, you know, things that we really need to do.	- That's one of the reasons why I can respond so strongly to this is because to me it's stupid to spend a lot of money and never ask the people that it's going to affect.
	- A lot of surveys, I participate in a lot of surveys and activities that provide feedback and information because I think it's critical to get it from shall we say the horses mouth instead of guessing for researchers.	- Yeah because like, it's like we're the ones that, we're the ones that are being you know, being worked on and so we should have some kind of say in what you're working on.	- If the focus of a research problem results in something that the people that are suffering from back pain won't do or isn't applicable to their daily life it won't be much value to them. If the fact that you could focus on something that people can actually do in their daily life to alleviate their discomfort is the most valuable thing. By asking them how likely are you to use any of the results along the way in the project I think will be very useful.
			- Well I certainly think somebody with back pain should be involved. Because otherwise you don't know what you're dealing with. I think you're right that is important, to have someone with back pain that

			maybe doesn't happen anymore but still can remember how awful it can be.
Tasks	- Well, they could, they ultimately could be one of the deciding factors as to whether something is funded or not. It's all eventually about whether you get funded.	- Maybe all patients should be given some kind of survey based on their level of injury. About their experiences with this and maybe things that they did that helped alleviate the pain besides what the doctor said. I don't know if that's a good answer but that's all I can come up with.	- By involving them as much as possible, even in framing the questions and the focus of the study. As I said, I think a lot of times research leaves out the very people that are most effected by the results of it. Anytime that they can be included from the very beginning of what's even going to be studied is important, and that along the way as well.
	- You can have a, probably a focus group. And there you get, you get a lot more development of ideas.	- I've had study questionnaires sent to me over the years. And they seem to work pretty well.	- Other than sending out questionnaires, maybe you should get them in a room. And talk to them in person
	- I think they could up the prioritizing the topics. Use those for actual quantified research that is you start to check back on the patients or the people in the study to see if the topics helped. For example, let's say the topic research was not providing more information on how back pain is caused. You know, if you start to adopt a topic, then check to see, provide information and then check to see from the people in the study how many of them felt that this improved the way of dealing with the back pain.	- Well, that's fairly, that's a tough one. I think probably the way you approached the focus group was the same approach you could use for that. And just be sure that depending on what type of research you're going to do that you get patients that are all on the same level.	
